# Southeast Atmosphere Studies: learning from model-observation syntheses

**DOI:** 10.5194/acp-18-2615-2018

**Published:** 2018-02-22

**Authors:** Jingqiu Mao, Annmarie Carlton, Ronald C. Cohen, William H. Brune, Steven S. Brown, Glenn M. Wolfe, Jose L. Jimenez, Havala O. T. Pye, Nga Lee Ng, Lu Xu, V. Faye McNeill, Kostas Tsigaridis, Brian C. McDonald, Carsten Warneke, Alex Guenther, Matthew J. Alvarado, Joost de Gouw, Loretta J. Mickley, Eric M. Leibensperger, Rohit Mathur, Christopher G. Nolte, Robert W. Portmann, Nadine Unger, Mika Tosca, Larry W. Horowitz

**Affiliations:** 1 Geophysical Institute and Department of Chemistry, University of Alaska Fairbanks, Fairbanks, AK, USA; 2 Department of Environmental Sciences, Rutgers University, New Brunswick, NJ, USA; 3 Department of Earth and Planetary Science, University of California, Berkeley, Berkeley, CA, USA; 4 Department of Meteorology, Pennsylvania State University, University Park, PA, USA; 5 Department of Chemistry and CIRES, University of Colorado Boulder, Boulder, CO, USA; 6 Cooperative Institute for Research in Environmental Sciences, University of Colorado, Boulder, Boulder, CO, USA; 7 Chemical Sciences Division, NOAA Earth System Research Laboratory, Boulder, CO, USA; 8 Joint Center for Earth Systems Technology, University of Maryland Baltimore County, Baltimore, MD, USA; 9 National Exposure Research Laboratory, US Environmental Protection Agency, Research Triangle Park, NC, USA; 10 School of Chemical and Biomolecular Engineering and School of Earth and Atmospheric Sciences, Georgia Institute of Technology, Atlanta, GA, USA; 11 Department of Chemical Engineering, Columbia University, New York, NY USA; 12 Center for Climate Systems Research, Columbia University, New York, NY, USA; 13 NASA Goddard Institute for Space Studies, New York, NY, USA; 14 Department of Earth System Science, University of California, Irvine, CA, USA; 15 Atmospheric and Environmental Research, Lexington, MA, USA; 16 John A. Paulson School of Engineering and Applied Sciences, Harvard University, Cambridge, MA, USA; 17 Center for Earth and Environmental Science, SUNY Plattsburgh, Plattsburgh, NY, USA; 18 College of Engineering, Mathematics and Physical Sciences, University of Exeter, Exeter, UK; 19 School of the Art Institute of Chicago (SAIC), Chicago, IL 60603, USA; 20 Geophysical Fluid Dynamics Laboratory–National Oceanic and Atmospheric Administration, Princeton, NJ, USA

## Abstract

Concentrations of atmospheric trace species in the United States have changed dramatically over the past several decades in response to pollution control strategies, shifts in domestic energy policy and economics, and economic development (and resulting emission changes) elsewhere in the world. Reliable projections of the future atmosphere require models to not only accurately describe current atmospheric concentrations, but to do so by representing chemical, physical and biological processes with conceptual and quantitative fidelity. Only through incorporation of the processes controlling emissions and chemical mechanisms that represent the key transformations among reactive molecules can models reliably project the impacts of future policy, energy and climate scenarios. Efforts to properly identify and implement the fundamental and controlling mechanisms in atmospheric models benefit from intensive observation periods, during which collocated measurements of diverse, speciated chemicals in both the gas and condensed phases are obtained. The Southeast Atmosphere Studies (SAS, including SENEX, SOAS, NOMADSS and SEAC4RS) conducted during the summer of 2013 provided an unprecedented opportunity for the atmospheric modeling community to come together to evaluate, diagnose and improve the representation of fundamental climate and air quality processes in models of varying temporal and spatial scales.

This paper is aimed at discussing progress in evaluating, diagnosing and improving air quality and climate modeling using comparisons to SAS observations as a guide to thinking about improvements to mechanisms and parameterizations in models. The effort focused primarily on model representation of fundamental atmospheric processes that are essential to the formation of ozone, secondary organic aerosol (SOA) and other trace species in the troposphere, with the ultimate goal of understanding the radiative impacts of these species in the southeast and elsewhere. Here we address questions surrounding four key themes: gas-phase chemistry, aerosol chemistry, regional climate and chemistry interactions, and natural and anthropogenic emissions. We expect this review to serve as a guidance for future modeling efforts.

## 1 Introduction

The southeastern US has been studied extensively because it includes intense emissions of biogenic volatile organic compounds (BVOCs; the definitions for the abbreviations used in this paper can be found in Appendix A) and has multiple large sources of anthropogenic emissions (e.g., Chameides et al., 1988; Trainer et al., 1987). An improved understanding of ozone photochemistry in this region has subsequently led to effective ozone control strategies (Council, 1991). In the 1990s, a number of aircraft and ground field campaigns were conducted to study ozone photochemistry in the southeastern US (Cowling et al., 2000, 1998; McNider et al., 1998; Hübler et al., 1998; Meagher et al., 1998; Martinez et al., 2003; Roberts et al., 2002; Stroud et al., 2001). Aggressive regulatory efforts over the past decade have substantially decreased NO*_x_* in this region (e.g., Russell et al., 2012). This decrease is changing the factors that control the NO*_x_* lifetime and offers an opportunity to study mechanisms of emission from ecosystems in the region in different chemical regimes. The decrease in NO*_x_* is also shifting the regime of HO*_x_* chemistry from one where the primary reaction partner for HO_2_ and RO_2_ was NO to one where isomerization, RO_2_+ HO_2_ and HO_2_+ HO_2_ are more important. The Southeast Atmosphere Studies (SAS, including SENEX, SOAS, NOMADSS and SEAC4RS), was designed to study the atmospheric chemistry of the region in the context of changing anthropogenic emissions.

Observational experiments in the southeastern US during SAS 2013 (SOAS, SENEX, SEAC4RS, NOMADSS) provide a wealth of new insights into the composition of the atmosphere. Results allow researchers to explore the chemical degradation of biogenic organic molecules over a range of concentrations of ambient nitrogen oxide during day and night and the ensuing consequences for ozone, aerosol and radiative properties of the atmosphere. The experiment was large and collaborative and included coordinated measurements at multiple surface sites and, among several aircraft, with many flyovers of the surface sites and a wide suite of available remote sensing from space-based instruments. A comprehensive array of instruments at each site or aircraft tracked most of the key atmospheric observables. Direct tracking of oxidative pathways was made possible by including gas-phase measurements of parent molecules and many of the first- and second-generation daughter molecules. For the first time, many of the daughter molecules were also tracked into the aerosol phase. These observations provided an important context for both the characterization of new instruments and new methods by interpreting measurements from more established instruments. In parallel with these field measurements, several laboratory experiments used the same instrumentation to provide insights into the chemical mechanisms of oxidation and instrument performance under field conditions. Overviews of the entire project and many of the subprojects have been presented elsewhere (Carlton et al., 2017; Warneke et al., 2016; Toon et al., 2016). Analyses of the observations have ranged from those that focus on the observations alone to those that primarily describe model simulations of the region. In this review we focus on the intersection of these two approaches, which is on analyses of observations that specifically test and inform the construction of 3-D chemical weather models. Our evaluations are focused on the southeast data set, although we assert that the lessons learned are global.

## 2 Gas-phase chemistry

### 2.1 Background

Global and regional models tend to significantly overestimate summertime surface ozone over the southeastern US (Fiore et al., 2009; Murazaki and Hess, 2006; Yu et al., 2010, 2007; Lin et al., 2008; Rasmussen et al., 2012), posing a challenge for air quality management in this region and elsewhere. It remains unclear whether this model bias in summertime surface ozone is mainly due to the chemical processes (e.g., HO*_x_* recycling, isoprene nitrate chemistry, heterogeneous reactions, nighttime chemistry), physical processes (e.g., dry deposition, boundary layer processes) or emissions. Fiore et al. (2005) suggested that this problem might be due to incorrect representation of isoprene sources and chemistry. Measured deposition rates for isoprene oxidation products appear to be higher than current model values (T. B. Nguyen et al., 2015; Karl et al., 2010). In the meantime, the understanding of isoprene oxidation chemistry has been evolving rapidly in the past decade (Crounse et al., 2011; Peeters et al., 2014, 2009), and as a result conclusions drawn from models using older chemical mechanism may not be correct.

A large debate surrounds our understanding of hydroxyl radical (OH) and hydroperoxy radical (HO_2_) concentrations in the presence of isoprene. Traditional mechanisms assume that isoprene oxidation suppresses OH concentrations in low-NO*_x_* conditions via the formation of organic hydroxyperoxides (Jacob and Wofsy, 1988). However, observations show higher-than-expected OH concentrations in isoprene-rich environments without corresponding enhancements in HO_2_ or RO_2_ (Tan et al., 2001; Carslaw et al., 2001; Lelieveld et al., 2008; Hofzumahaus et al., 2009; Ren et al., 2008; Pugh et al., 2010; Thornton et al., 2002; Stone et al., 2010), suggesting a gap in current understanding of isoprene oxidation. On the other hand, an interference has been discovered to affect some of these OH instruments (Mao et al., 2012; Novelli et al., 2014; Feiner et al., 2016).

Measurements of higher-than-expected OH in the presence of isoprene spurred renewed interest in issues related to the products of the HO_2_+ RO_2_ reactions. Thornton et al. (2002) and Hasson et al. (2004) had pointed out that if this reaction does not terminate the radical chain it would change the behavior of HO*_x_* radicals at low NO*_x_*. Several specific cases of the HO_2_+ RO_2_ reactions were shown to have an OH product (Hasson et al., 2004; Jenkin et al., 2007; Dillon and Crowley, 2008). Peeters et al. (2009, 2014) identified a new path for OH regeneration through unimolecular isomerization of isoprene hydroxyperoxy radicals. This pathway was confirmed by laboratory measurements of its rate (Crounse et al., 2011; Teng et al., 2017). A key feature of the SAS experiments was that the NO*_x_* concentrations spanned a range that resulted in measurements where the three major fates of isoprene peroxy radicals (reaction with NO, HO_2_ or isomerization) were sampled at different times and locations.

Another major consequence of isoprene oxidation is the production of isoprene nitrates, formed from RO_2_+NO reaction in the isoprene degradation chain during daytime and by addition of NO_3_ to the double bonds in isoprene or isoprene daughters at night. Different treatments of these reactions in models including the yield and subsequent fate of daytime isoprene nitrates cause as much as 20 % variation in global ozone production rate and ozone burden among different models (Ito et al., 2009; Horowitz et al., 2007; Perring et al., 2009a; Wu et al., 2007; Fiore et al., 2005; Paulot et al., 2012). Large variations mainly stem from the different yield of isoprene nitrates (Wu et al., 2007) and the NO*_x_* recycling ratio of these isoprene nitrates (Ito et al., 2009; Paulot et al., 2012). Recent laboratory data indicates the yield of first-generation isoprene nitrates is in the range of 9 to 14 % (Giacopelli et al., 2005; Patchen et al., 2007; Paulot et al., 2009a; Lockwood et al., 2010; Sprengnether et al., 2002; Xiong et al., 2015; Teng et al., 2015), which is much higher than the 4 % that was suggested as recently as 2007 (Horowitz et al., 2007). The subsequent fate of these isoprene nitrates includes oxidation by OH, NO_3_ and O_3_ (Lockwood et al., 2010; Paulot et al., 2009a; Lee et al., 2014); photolysis (Müller et al., 2014); and hydrolysis. Synthesis of models and SAS observations suggest an important role for hydrolysis as expected based on the laboratory measurements (Romer et al., 2016; Fisher et al., 2016; Wolfe et al., 2015).

The SAS observations also provide measurements that guide our thinking about the role of NO_3_ chemistry and its representation in models, especially as it contributes to oxidation of biogenic volatile organic compounds at night (Warneke et al., 2004; Brown et al., 2009; Aldener et al., 2006; Ng et al., 2008, 2017; Edwards et al., 2017). During SAS, these reactions were a substantial sink of NO*_x_* in addition to their role in oxidation of BVOCs. To a large extent this is due to the high yield of carbonyl nitrates (65–85 %) from the isoprene + NO_3_ oxidation (Perring et al., 2009b; Rollins et al., 2009, 2012; Kwan et al., 2012; Schwantes et al., 2015). Models that incorporate this chemistry (Xie et al., 2013; Horowitz et al., 2007; von Kuhlmann et al., 2004; Mao et al., 2013) indicate that the isoprene + NO_3_ reaction contributes more than 50 % of the total isoprene nitrate production and that the reaction is thus a major pathway for nighttime NO*_x_* removal. The fate of products from isoprene + NO_3_ and to what extent they return NO*_x_* remains a subject of discussion and thus an opportunity for exploration with models that might guide our thinking about a plausible range of product molecules (Perring et al., 2009b; Müller et al., 2014; Schwantes et al., 2015).

Compared to isoprene, the oxidation mechanism of monoterpene has received much less attention partly due to lack of laboratory and field data. In contrast to isoprene, a significant portion of terpenes emissions is released at night. Browne et al. (2014) showed that monoterpene oxidation is a major sink of NO*_x_* in the Arctic. The high yield of organic nitrates (ONs) and the low vapor pressure and high solubility of monoterpene organic nitrates result in strong coupling of gas-phase mechanisms to predictions of secondary organic aerosol (SOA) in a model. For example, the reaction of terpenes + NO_3_ provides a large source of SOA as inferred (Ng et al., 2017). These aerosol organic nitrates can be either a permanent or temporary NO*_x_* sink depending on their precursors as well as ambient humidity (Nah et al., 2016b; Boyd et al., 2015; B. H. Lee et al., 2016; Romer et al., 2016). Some of the monoterpene organic nitrates may be susceptible to rapid hydrolysis and photolysis in aerosol phase (thus not detected as aerosol nitrates), leading to an underestimate of its contribution to SOA mass (Rindelaub et al., 2015, 2016).

### 2.2 Major relevant findings

A major focus of the SAS study was to study the daytime and nighttime oxidative chemistry of isoprene and to compare the observations against models representing the ideas outlined above. Over the range of the fate of the isoprene RO_2_ radical, isomerization was important and the reaction partners were mostly NO and HO_2_ during the day and a mix of NO_3_, RO_2_ and HO_2_ at night. The field measurements were closely partnered with laboratory chamber experiments (Nguyen et al., 2014b) which enhanced our understanding of oxidation mechanisms and provided increased confidence in our understanding of the measurements of isoprene oxidation products. We summarize these major relevant findings as follows.

1. Radical simulation: combining traditional laser-induced fluorescence with a chemical removal method that mitigates potential OH measurement artifacts, Feiner et al. (2016) found that their tower-based measurements of OH and HO_2_ during SOAS show no evidence for dramatically higher OH than current chemistry predicts in an environment with high BVOCs and low NO*_x_*. Instead, they are consistent with the most up-to-date isoprene chemical mechanism. Their measurements are also in agreement with collocated OH measurements by another technique, chemical ionization mass spectrometry (CIMS; Sanchez et al., 2017). Romer et al. (2016) found that the lifetime of NO*_x_* was consistent with these OH observations and that the major source of HNO_3_ was isoprene nitrate hydrolysis. Their conclusions would be inconsistent with dramatically higher OH levels, which would imply much more rapid isoprene nitrate production than observed. Other ratios of parent and daughter molecules and chemical lifetimes are also sensitive to OH and these should be explored for additional confirmation or refutation of ideas about OH production at low NO*_x_*.
  Isoprene vertical flux divergence in the atmospheric boundary layer over the SOAS site and similar forest locations was quantified by Kaser et al. (2015) during the NSF/NCAR C-130 aircraft flights and used to estimate daytime boundary layer average OH concentrations of 2.8 to 6.6 × 10^6^ molecules cm^−3^. These values, which are based on chemical budget closure, agree to within 20 % of directly observed OH on the same aircraft. After accounting for the impact of chemical segregation, Kaser et al. (2015) found that current chemistry schemes can adequately predict OH concentrations in high-isoprene regimes. This is also consistent with the comparison between measured and modeled OH reactivity on a ground site during SOAS, which show excellent agreement above the canopy of an isoprene-dominated forest (Kaiser et al., 2016).
2. Isoprene oxidation mechanism: recent refinements in our understanding of the early generations of isoprene degradation have stemmed from a synergy of laboratory, field, and modeling efforts. Laboratory work has provided constraints on the production and fate of a wide range of intermediates and end products, including organic nitrates (Teng et al., 2015; Xiong et al., 2015; Lee et al., 2014; Müller et al., 2014), the isoprene RO_2_ (Teng et al., 2017), IEPOX (St. Clair et al., 2015; Bates et al., 2014, 2016), MVK (methyl vinyl ketone; Praske et al., 2015) and MACR (methacrolein; Crounse et al., 2012). These experiments have been guided and/or corroborated by analyses of field observations of total and speciated alkyl nitrates (Romer et al., 2016; T. B. Nguyen et al., 2015; Xiong et al., 2015; B. H. Lee et al., 2016), IEPOX / ISOPOOH (isoprene hydroxy hydroperoxide; T. B. Nguyen et al., 2015), glyoxal (Min et al., 2016), HCHO (Wolfe et al., 2016), OH reactivity (Kaiser et al., 2016) and airborne fluxes (Wolfe et al., 2015). Recent modeling studies have incorporated these mechanisms to some extent and showed success on reproducing temporal and spatial variations of these compounds (Su et al., 2016; Fisher et al., 2016; Travis et al., 2016; Zhu et al., 2016; Li et al., 2018, 2016), as summarized in Table 1. Continued efforts are needed to reduce new-found mechanistic complexity for inclusion in regional and global models.
3. Oxidized VOC: large uncertainties remain on the production of smaller oxidation products. Several modeling studies indicate an underestimate of HCHO from isoprene oxidation in current mechanisms (Wolfe et al., 2016; Li et al., 2016; Marvin et al., 2017). Current chemical mechanisms differ greatly on the yield of glyoxal from isoprene oxidation (Li et al., 2016; Chan Miller et al., 2017). The observations indicate that the ratio of glyoxal to HCHO is 2 %, independent of NO*_x_* (Kaiser et al., 2015), and this ratio is reproduced, at least to some extent, in two modeling studies (Li et al., 2016; Chan Miller et al., 2017). Confirmation of such a ratio is a useful indicator as these molecules are also measured from space and both are short-lived and tightly coupled to oxidation chemistry. Widespread ambient confirmation of the ratio is difficult because of large biases in satellite glyoxal quantification (Chan Miller et al., 2017).
  For the case of the major daughter products methyl vinyl ketone and methacrolein, lab experiments have confirmed that ambient measurements reported to be MVK and MACR, by instruments with metal inlets including gas chromatography (GC) and proton-transfer-reaction mass spectrometry (PTR-MS), are more accurately thought of as a sum of MVK, MACR and isoprene hydroperoxides that react on metal and are converted to MVK and MACR (Rivera-Rios et al., 2014; Liu et al., 2013).
4. Organic Nitrates: the assumed lifetime and subsequent fate of organic nitrates can profoundly influence NO*_x_* levels across urban–rural gradients (Browne and Cohen, 2012; Mao et al., 2013), affecting oxidant levels and formation of secondary organic aerosol. Field observations during SAS suggest a short (2–3 h) lifetime of total and isoprene and terpene organic nitrates (Wolfe et al., 2015; Romer et al., 2016; Fisher et al., 2016; B. H. Lee et al., 2016). One possible explanation is aerosol uptake of these organic nitrates followed by rapid hydrolysis as confirmed in laboratory experiments (Hu et al., 2011; Darer et al., 2011; Rindelaub et al., 2016, 2015; Jacobs et al., 2014; Bean and Hildebrandt Ruiz, 2016), although the hydrolysis rate varies greatly with the structure of nitrate and aerosol acidity (Hu et al., 2011; Rindelaub et al., 2016; Boyd et al., 2017, 2015).
5. Nighttime chemistry: the SAS studies examined nighttime BVOC oxidation in both the nocturnal boundary layer (NBL) and the residual layer (RL). Measurements at the SOAS ground site provided a wealth of detailed information on nighttime oxidation processes in the NBL via state-of-the-art instrumentation to constrain the major oxidants, BVOCs and gas- and aerosol-phase products (Ayres et al., 2015; Xu et al., 2015b; B. H. Lee et al., 2016). A major focus of these efforts was to understand the influence of nitrate radical (NO_3_) oxidation as a source of secondary organic aerosol. These results are reviewed in Sect. 3.2.3 below and show that organic nitrates from reactions of NO_3_ with monoterpenes are an important SOA source in the NBL. Reactions of monoterpenes dominate nighttime chemistry near the surface due to their temperature-dependent (but not sunlight-dependent) emissions and their accumulation to higher concentration in the relatively shallow NBL.
  Nighttime flights of the NOAA P-3 probed the composition of the overlying RL and the rates of nighttime oxidation processes there. In contrast to the NBL, isoprene dominates the composition of BVOCs in the RL, with mixing ratios over Alabama on one research flight demonstrating a nighttime average near 1 ppbv. Monoterpene mixing ratios were more than an order of magnitude lower. Consumption of isoprene by O_3_ and NO_3_ was shown to depend on the sunset ratio of NO*_x_* to isoprene, with NO_3_ reaction dominating at ratios above approximately 0.5 and O_3_ reaction dominant at lower ratios. Overall, O_3_ and NO_3_ contributed approximately equally to RL isoprene oxidation in the 2013 study. This observation, combined with recent trends in NO*_x_* emissions, suggests that RL nighttime chemistry in the southeastern US is currently in transition from a NO*_x_* -dominated past to an O_3_-dominated future, a condition more representative of the preindustrial past. The implications of this trend for understanding organic nitrates and secondary organic aerosol should be considered in models of the influence of changing NO*_x_* emissions on BVOC oxidation (Edwards et al., 2017).
6. HONO: the community’s confusion about sources of HONO was not resolved by SAS. Airborne observations over water from the NCAR C-130 suggest that conversion of HNO_3_ to HONO and NO*_x_* via photolysis of particulate nitrate in the marine boundary layer is important (Ye et al., 2016). A separate study using NOAA WP-3D observations indicates that HONO mixing ratios in the background terrestrial boundary layer are consistent with established photochemistry (Neuman et al., 2016). Persistent uncertainties regarding the potential for measurement artifacts continue to hamper efforts to resolve outstanding questions about putative novel HONO sources.
7. Higher-order terpenes: monoterpene and sesquiterpene chemistry requires continued investigation. Initial studies indicate that monoterpene oxidation can be an important sink of NO*_x_* and an important source of aerosol precursors (B. H. Lee et al., 2016; Ayres et al., 2015). Additional analysis is needed to understand the role of monoterpenes. We note that because our understanding of isoprene chemistry has been changing so rapidly and because the role of isoprene sets the stage for evaluating the role of monoterpenes, we are now in a much better position to evaluate the role of monoterpene chemistry.

### 2.3 Model recommendations

**Figure 1:**
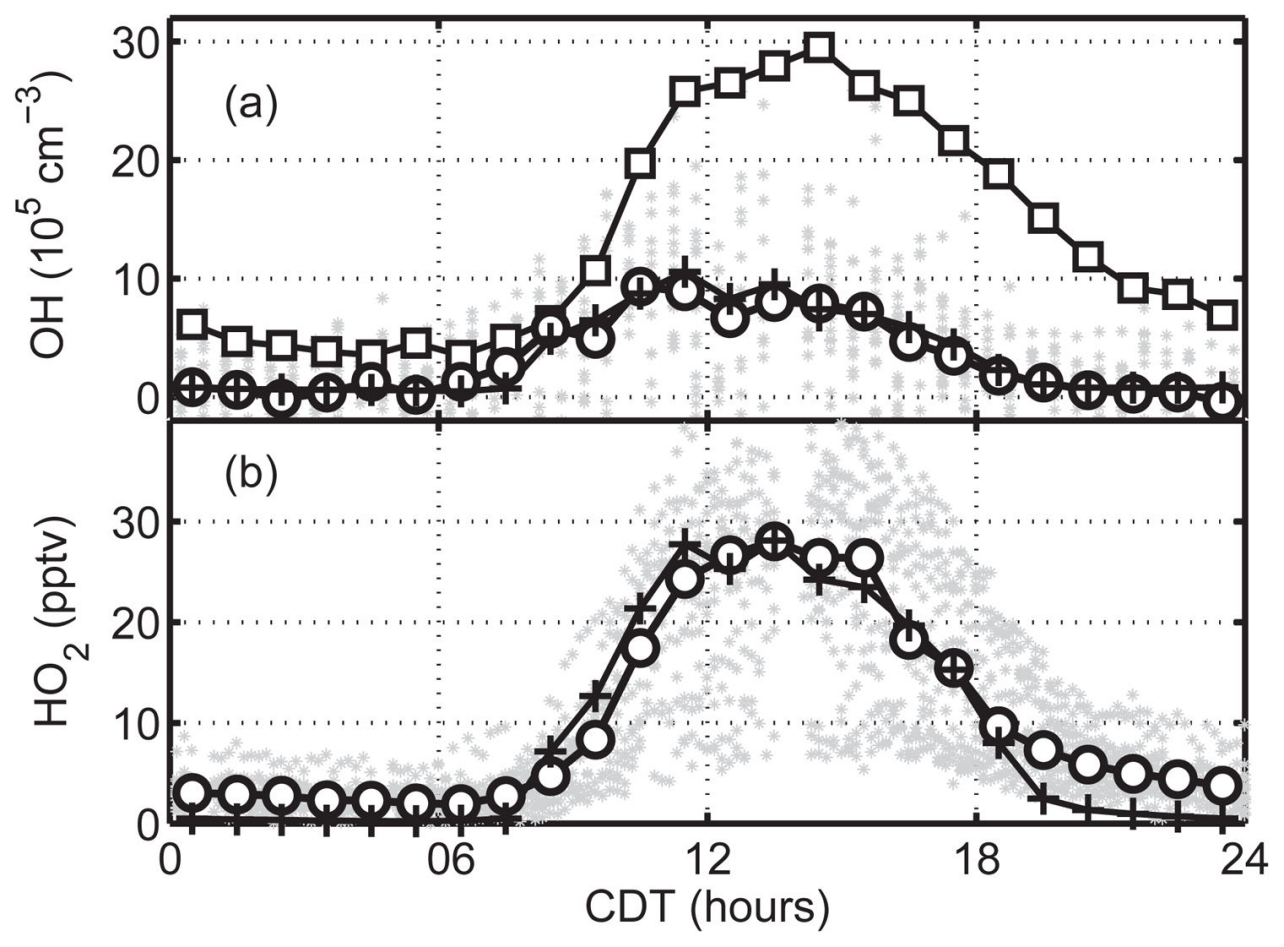
Diel variation of measured and modeled OH / HO_2_ during SOAS (Feiner et al., 2016). In panel **(a)**, measured OH by a traditional laser-induced fluorescence technique is shown in squares and by a new chemical scavenger method is shown in circles. The latter one is considered as the “true” ambient OH. Simulated OH from a photochemical box model with Master Chemical Mechanism (MCM) v3.3.1 is shown in pluses. In panel **(b)**, measured HO_2_ is shown in circles and modeled HO_2_ is shown in pluses. For both panels, gray dots are individual 10 min measurements.

Based upon the improved understanding outlined above, we make the following recommendations for the future modeling efforts:

1. Measurements and modeling effort on OH show no indication of a need for empirical tuning factors to represent OH chemistry in the rural southeastern US. Detailed mechanisms based on recent laboratory chamber studies (mostly at Caltech) and theoretical studies (Leuven) for isoprene result in predicted OH that is in reasonable agreement with observations (Fig. 1). Condensed mechanisms that approximate the detailed ones are expected to do the same. Whatever mechanism is used, a key diagnostic identified is the parent–daughter molecular relationships such as NO_2_ / HNO_3_ or MVK / isoprene. Models calculations should emphasize opportunities for observations of such ratios as an independent measure of the effect of OH on the atmosphere.
2. The chemistry of isoprene should be treated in more detail than most other molecules. We recommend that there should be explicit chemistry through the first and second generation of isoprene oxidation to better illustrate the role of isoprene in ozone production, OH budget and SOA production. No other species should be lumped with isoprene or its daughters. Even for climate models that cannot afford this level of complexity, a reduced mechanism of isoprene oxidation should be generated for a wide range of conditions.
3. NO_3_ chemistry is an important element of VOC oxidation, NO*_x_* removal and aerosol production. NO_3_ chemistry should be included in models that do not explicitly take it into account, both as a loss process of VOCs and NO*_x_* and as a source of aerosols.
4. The largest NO*_x_* and BVOC emissions are not collocated, as one is mainly from mobile sources and power plants and the other one is mainly from forests (Yu et al., 2016; Travis et al., 2016). As a result, model resolution can impact predicted concentrations of trace species. Different model resolutions may lead to as much as 15 % differences at the tails of the NO*_x_* and HCHO distribution – less so for O_3_ (Yu et al., 2016; Valin et al., 2016). Depending on the research question, models should evaluate the need to resolve this last 15 %, which requires a horizontal resolution of order 12 km or less.

### 2.4 Key model diagnostics

We identified a number of key diagnostics that should probably be evaluated before a model is used to pursue more interesting new questions. These include the following.

1. NO*_x_* concentrations from in situ and satellite observations. Models that do not predict the correct magnitude of NO*_x_* should produce the wrong OH, O_3_ and parent : daughter VOC ratios (e.g., isoprene : isoprene + IEPOX, isoprene : MACR + MVK). At the low-NO*_x_* characteristic of the southeastern US these errors are approximately linear – that is, a 15 % error in NO*_x_* should correspond to a 15 % error in OH, isoprene and other related species. Given the difficulty in predicting NO*_x_* to this tolerance, caution should be taken not to over-interpret model predictions.
2. HCHO from space-based observations is emerging as a useful diagnostic of model oxidation chemistry (Valin et al., 2016).
3. A significant fraction of isoprene remains at sunset and is available for oxidation via O_3_ or NO_3_ at night. Analysis of nighttime isoprene and its oxidation products in the RL in the northeast US in 2004 suggested this fraction to be 20 % (Brown et al., 2009). Preliminary analysis from SENEX suggested a similar fraction, although the analysis depends on the emission inventory for isoprene, and would be 10–12 % if isoprene emissions were computed from MEGAN (see Sect. 4.2 for the difference between BEIS and MEGAN). This fact might be a useful diagnostic of boundary layer dynamics and nighttime chemistry in models. The overnight fate of this isoprene depends strongly on available NO*_x_* (see above). More exploration of the model prediction of the products of NO_3_+ isoprene and additional observations of those molecules will provide insight into best practices for using it as a diagnostic of specific model processes.
4. O_3_ and aerosol concentrations and trends over decades and contrasts between weekdays and weekends across the southeast remain a valuable diagnostic of model performance, especially as coupled to trends in NO*_x_* on those same timescales.

### 2.5 Open questions

There are many open questions related to gas-phase chemistry. Here we highlight a few that we believe are best addressed by the community of experimentalists and modelers working together (there were many other open questions that we think could be addressed by individual investigators pursuing modeling or experiments on their own).

1. The sources and sinks of NO*_x_* are not well constrained in rural areas that cover most of the southeastern US. As anthropogenic-combustion-related emissions experience further decline, what do we expect to happen to NO*_x_*? What observations would test those predictions?
2. As we are reaching consensus on a mechanism for isoprene oxidation, the role of monoterpene and sesquiterpene oxidation is becoming a larger fraction of remaining uncertainty. Strategies for exploring and establishing oxidation mechanisms for these molecules and for understanding the level of detail needed in comprehensive and reduced mechanisms are needed.
3. Air quality modeling efforts have long been most interested in conditions that are not of top priority to meteorological researchers – e.g., stagnation. In addition to a better understanding of horizontal flows in stagnant conditions these experiments highlighted the need for a deeper understanding of the links between chemical mixing and boundary layer dynamics in day and night. A number of new chemical observations have been identified in the southeastern US data sets. Combined approaches using models and these observations to guide our thinking about planetary boundary layer (PBL) dynamics are needed.

## 3 Organic aerosol

### 3.1 Background

Improving the representation of organic aerosol (OA) is a critical need for models applied to the southeast. Current air quality and chemistry–climate models produce a very wide range of organic aerosol mass concentrations, with predicted concentrations spread over 1–2 orders of magnitude in free troposphere (Tsigaridis et al., 2014). Secondary OA (SOA) has traditionally been modeled by partitioning of semivolatile species between the gas and aerosol phase (Odum et al., 1996; Chung and Seinfeld, 2002; Farina et al., 2010), but very large uncertainties remain on the detailed formulations implemented in models (Spracklen et al., 2011; Heald et al., 2011; Tsigaridis et al., 2014). In particular, the recent identification of substantial losses of semivolatile and intermediate volatility species to Teflon chamber walls (Matsunaga and Ziemann, 2010; Zhang et al., 2014; Krechmer et al., 2016; Nah et al., 2016a) necessitates a re-evaluation of the gas-phase SOA yields used in models which has yet to be comprehensively performed. Models have difficulties in reproducing the mass loading of OA in both urban and rural areas, although order-of-magnitude underestimates have only been observed consistently for urban pollution (e.g., Volkamer et al., 2006; Hayes et al., 2015). Furthermore, current OA algorithms often rely on highly parameterized empirical fits to laboratory data that may not capture the role of oxidant (OH vs. O_3_ vs. NO_3_) or peroxy radical fate. The peroxy radical fate for historical experiments, in particular, may be biased compared to the ambient atmosphere where peroxy radical lifetimes are longer and autoxidation can be important.

Recent laboratory, field and model studies suggest that a significant fraction of SOA is formed in aqueous-phase cloud droplets and aerosols, following gas-phase oxidation to produce soluble species (Sorooshian et al., 2007; Fu et al., 2008; Myriokefalitakis et al., 2011; Carlton et al., 2008; Tan et al., 2012; Ervens et al., 2011; Volkamer et al., 2009). This is also consistent with the strong correlation between OA and aerosol liquid water in the southeastern US over the past decade (T. K. V. Nguyen et al., 2015). A number of gas-phase VOC oxidation products have been recognized as important precursors for aqueous production of SOA, including epoxides (Pye et al., 2013; Nguyen et al., 2014a; Surratt et al., 2010) and glyoxal (Liggio et al., 2005; Woo and McNeill, 2015; McNeill et al., 2012). Aerosol uptake of these oxygenated VOCs can be further complicated by aerosol acidity and composition (Pye et al., 2013; Paulot et al., 2009b; Nguyen et al., 2014a; Marais et al., 2016).

While a significant portion of ambient OA has been attributed to various source classes and precursors (e.g., BBOA from biomass burning; IEPOX-SOA from isoprene epoxydiols or IEPOX; and less-oxidized oxygenated OA, LO-OOA, from monoterpenes), a large portion of ambient OA (e.g., more-oxidized oxygenated OA, MO-OOA) remains unapportioned. This portion lacks detailed chemical characterization or source attribution, so further investigation is warranted (Xu et al., 2015b, a). A diversity of modeling approaches, including direct scaling with emissions, reactive uptake of gaseous species and gas–aerosol partitioning, is encouraged to provide insight into OA processes while trying to make use of all available experimental constraints to evaluate the models.

### 3.2 Major relevant findings

A number of modeling groups will be interested in modeling aerosol for the Southeast Atmosphere Study across a variety of spatial and temporal scales. Different studies will be able to support different levels of detail appropriate for their application. Detailed box-model representations can serve to confirm or refute mechanisms and, eventually, be condensed for application at larger scales such as those in chemical transport (CTM) or general circulation (GCM) models. In the following sections, we highlight areas of organic aerosol that should be represented.

#### 3.2.1 Partitioning theory and phases

No large kinetic limitations to partitioning are observed in the southeast, and partitioning according to vapor pressure is active on short timescales (Lopez-Hilfiker et al., 2016). The higher relative humidity (RH) in this region, which results in fast diffusion in isoprene-SOA containing particles (Song et al., 2015), may be at least partially responsible for this behavior. In some instances (e.g., for key IEPOX-SOA species), observations indicate that detected OA species are significantly less volatile than their structure indicates, likely due to thermal decomposition of their accretion products or inorganic–organic adducts in instruments (Lopez-Hilfiker et al., 2016; Hu et al., 2016; Isaacman-VanWertz et al., 2016; Stark et al., 2017).

Further research is needed regarding the role of organic partitioning into OA versus water and this can be evaluated using field data. If both processes occur in parallel in the atmosphere, vapor-pressure-dependent partitioning to OA may occur along with aqueous processing without significant double counting or duplication in models. However, due to the high relative humidity (average RH is 74 %, see Weber et al., 2016) and degree of oxygenation of organic compounds (OM / OC is 1.9–2.25, see below) in the southeastern US atmosphere, inorganic-rich and organic-rich phases may not be distinct (You et al., 2013) and more advanced partitioning algorithms accounting for a mixed inorganic–organic water phase may be needed (Pye et al., 2017, 2018).

Phase separation can be predicted based on the determination of a separation relative humidity (SRH), which is a function of the degree of oxygenation and inorganic constituent identity (You et al., 2013), and a comparison to the ambient relative humidity. For RH < SRH, phase separation occurs. Pye et al. (2017) predicted phase separation into organic-rich and electrolyte-rich phases occurs 70 % of the time during SOAS at CTR with a higher frequency during the day due to lower RH.

#### 3.2.2 Primary organic aerosol

Primary organic aerosol (POA) concentrations are expected to be small in the southeast outside urban areas and we make no major recommendation for how to model them. Modelers should be aware that a fraction of primary organic aerosol based on the EPA National Emissions Inventory (NEI) is semivolatile (Robinson et al., 2007). However, not all POA is thought to be semivolatile – for example, OAs from sources such as soil are included in the NEI. Modeled POA may already include some oxidized POA (OPOAs) if the models include heterogeneous oxidation (as in CMAQ; Simon and Bhave, 2012) or hydrophilic conversion (as in GEOS-Chem; Park et al., 2003). Thus, care should be exercised in evaluating model species such as POA with aerosol mass spectrometer (AMS) positive matrix factorization (PMF) factors such as hydrocarbon-like OA (HOA). For semivolatile POA treatments, mismatches between POA inventories and semivolatile / intermediate volatility organic compounds (S / IVOCs) need to be carefully considered. Comparisons of model inventory versus ambient ratios of POA / ΔCO, POA / black carbon (BC) or POA / NO*_x_* can be used to indicate whether or not POA emissions are excessive (De Gouw and Jimenez, 2009). As these ratios can be affected by errors in the denominator species, it is important to also evaluate those carefully against observations. For models with limited POA information, the ratio of organic matter to organic carbon (OM / OC) should be adjusted to reflect the highly oxidized nature of ambient OA (as mass is transferred from hydrophobic/hydrophilic concentrations for example). The OM / OC ratio of bulk ambient OA in the southeastern US is 1.9–2.25 as measured during summer 2013 (Kim et al., 2015; Pye et al., 2017).

A biomass burning PMF factor (BBOA) was observed during SOAS and likely has a higher impact on brown carbon (BrC) than its contribution to OA mass would suggest, although overall BrC concentrations were very small (Washenfelder et al., 2015). Net SOA mass added via photochemical processing of biomass burning emissions is thought to be modest, relative to the high POA emissions (Cubison et al., 2011; Jolleys et al., 2012; Shrivastava et al., 2017).

#### 3.2.3 Particle-phase organic nitrates

Organic nitrates, primarily from monoterpene reactions with the nitrate radical, have been recognized as an important source of OA in the southeast, contributing from 5 to 12 % in the southeastern US in summer (Xu et al., 2015a, b; Ayres et al., 2015; Pye et al., 2015; B. H. Lee et al., 2016). In fact, this number could be an underestimate if some of these organic nitrates are susceptible to hydrolysis or photodegradation and thus are not detected as nitrates. We have high confidence that models should include SOA formation from nitrate radical oxidation of monoterpenes. Sesquiterpenes and isoprene may also contribute OA through nitrate radical oxidation, but the contribution is expected to be smaller (Pye et al., 2015; Fisher et al., 2016). A number of options exist for representing this type of aerosol including fixed yields, Odum 2-product parameterizations, volatility basis set (VBS) representations (Boyd et al., 2015) and explicit partitioning and/or uptake of organic nitrates (Pye et al., 2015; Fisher et al., 2016).

Detailed modeling studies can provide additional insight into the interactions between monoterpene nitrate SOA and gas-phase chemistry, as well as the fates of specific organic nitrates. Explicit formation and treatment of organic nitrates, yields of which are parent hydrocarbon specific, can take into account hydrolysis of particle-phase organic nitrate. The hydrolysis should depend on the relative amounts of primary, secondary and tertiary nitrates which are produced in different abundances in photooxidation vs. nitrate radical oxidation (Boyd et al., 2015, 2017). Hydrolysis may also depend on the level of acidity and presence of double bonds in the organic nitrate (Jacobs et al., 2014; Rindelaub et al., 2016). In addition to hydrolysis, particle organic nitrates could photolyze and release NO*_x_* or serve as a NO*_x_* sink through deposition (Nah et al., 2016b).

Formation of organic nitrates should also be considered in the context of emerging evidence for the role of autoxidation, especially in the monoterpene system (Ehn et al., 2014). Autoxidation has been shown to occur in both photooxidation and ozonolysis of monoterpenes (Jokinen et al., 2015) and leads to highly oxidized species including organic nitrates (B. H. Lee et al., 2016; Nah et al., 2016b), many of which are low volatility. While some empirical representations (e.g., VBS or Odum 2-product) of monoterpene SOA may capture these species, autoxidation products may be very susceptible to chamber wall loss (Zhang et al., 2014; Krechmer et al., 2016) and missing from SOA parameterizations. The role of autoxidation in forming SOA in the southeastern US atmosphere remains to be determined. In this regard, future laboratory studies should carefully constrain the peroxy radical reaction channels (e.g., Schwantes et al., 2015; Boyd et al., 2015) and be conducted under regimes that are representative of ambient environments where the peroxy radical lifetimes can vary.

#### 3.2.4 Isoprene epoxydiol (IEPOX)-SOA

Due to the abundance of observations in the southeastern atmosphere (Budisulistiorini et al., 2016; W. W. Hu et al., 2015; Hu et al., 2016; Xu et al., 2015a, b, 2016), similarity between laboratory and field IEPOX-SOA determined by PMF analysis and availability of model parameterizations to predict IEPOX-SOA (Pye et al., 2013; Woo and McNeill, 2015; Marais et al., 2016; Budisulistiorini et al., 2017; Sareen et al., 2017), we have high confidence that IEPOX-SOA should be included in models. D’Ambro et al. (2017) predicts IEPOX will be the major precursor to SOA under low-NO*_x_* conditions when peroxy radical lifetimes are atmospherically relevant, which has not always been the case in older experiments. However, a number of parameters needed to predict IEPOX-SOA are uncertain and different modeling approaches, as well as the use of all available experimental constraints, could be beneficial. The mechanism of IEPOX-SOA formation involves gas-phase reactions followed by aqueous processing which can occur either in aerosols or cloud droplets, although the acid-catalyzed initiation step of the epoxide ring opening favors SE US aerosol conditions and makes this process less efficient in cloud water. This mechanism could be represented as heterogeneous reaction with a reactive uptake coefficient or more explicit partitioning and particle reaction (Table 1).

**Figure 2:**
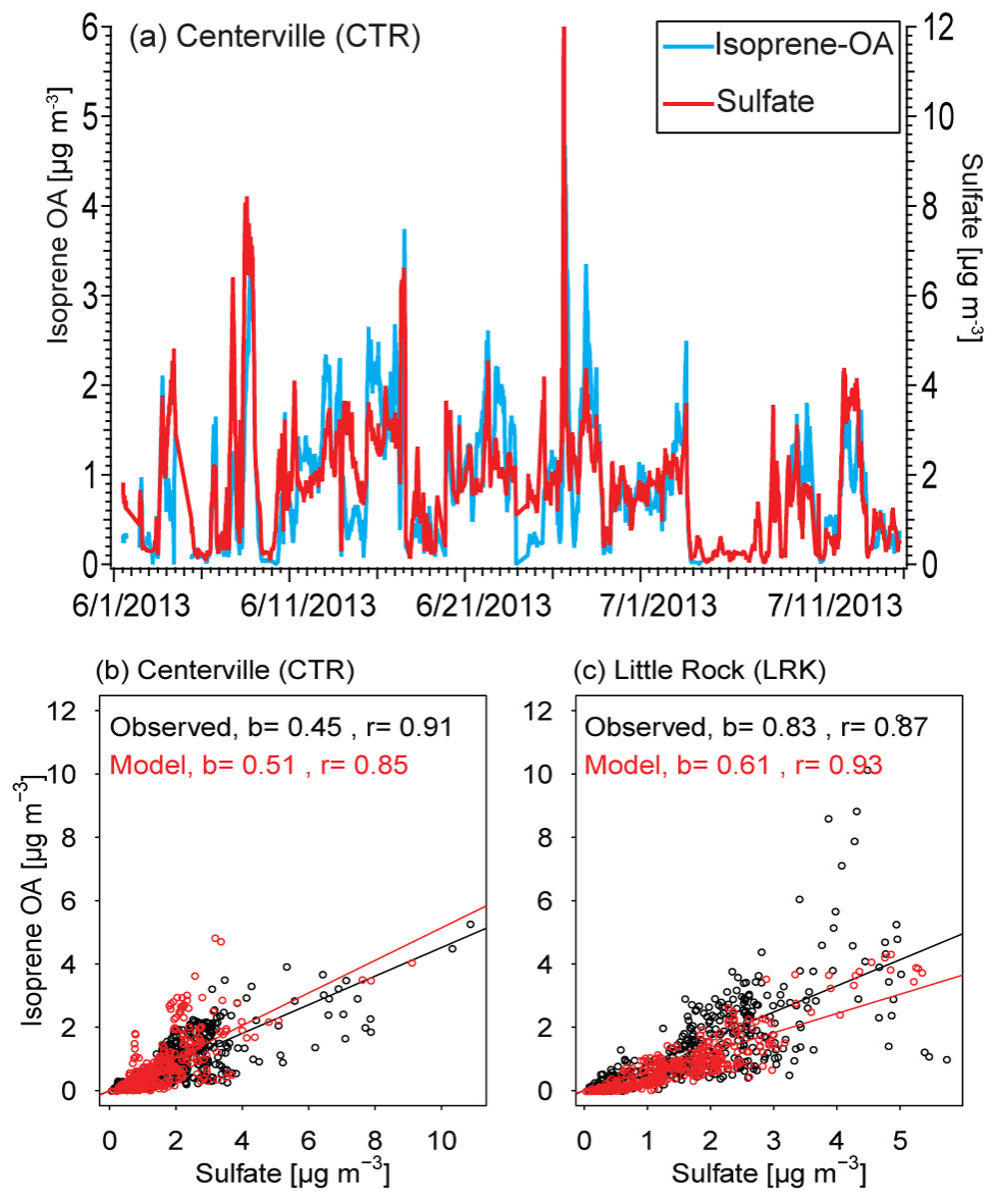
Time series and correlation between isoprene OA and sulfate during SOAS (Pye et al., 2016; Xu et al., 2015). Panel **(a)** shows the time series of both isoprene OA and sulfate at the Centerville site during SOAS. Panel **(b)** and **(c)** shows the correlation plot between isoprene OA and sulfate from both measurements and model results at two sites (Centerville and Little Rock) during SOAS.

The correlation of IEPOX-SOA with sulfate (Xu et al., 2015a, 2016; W. W. Hu et al., 2015) can serve as a useful model evaluation technique as underestimates in sulfate could lead to underestimates in IEPOX-SOA in models (Fig. 2). Current pathways for IEPOX-SOA formation (Eddingsaas et al., 2010) involve acidity in aqueous solutions (Kuwata et al., 2015), but several studies suggest that IEPOX-SOA is not correlated well with aerosol acidity or aerosol water (Budisulistiorini et al., 2017; Xu et al., 2015a). Ion balances or other simple measures of aerosol acidity are likely inadequate to characterize particle acidity and thermodynamic models such as ISORROPIA II or AIM are more appropriate for modeling IEPOX-SOA (Guo et al., 2015; Weber et al., 2016). Currently, different observational data sets indicate different nominal ratios of ammonium to sulfate (Pye et al., 2018), so it needs to be kept in mind that some measurements report only inorganic sulfate (e.g., ion chromatography) while others report total (inorganic + organic) sulfate (e.g., AMS). A modeling study suggested that ammonia uptake might be limited by organics, thus affecting acidity (Kim et al., 2015; Silvern et al., 2017).

SAS observations also provide estimates of some components of IEPOX-SOA including 2-methyltetrols and IEPOX–organosulfates (Budisulistiorini et al., 2015; W. W. Hu et al., 2015). For modeling applications focusing on IEPOX-SOA, additional speciation of IEPOX-SOA (into tetrols, organosulfates, etc.) and oligomerization and volatility can be treated. Treating the monomers (e.g., 2-methyltetrols) explicitly with their molecular properties will likely lead to excessive volatility of the IEPOX-SOA (Lopez-Hilfiker et al., 2016; Hu et al., 2016; Isaacman-VanWertz et al., 2016; Stark et al., 2017).

#### 3.2.5 Glyoxal SOA

New information on glyoxal SOA is emerging in this area but its importance in the southeast remains unclear. Glyoxal has been suspected to be the dominant aqueous SOA source under high-NO*_x_* (RO_2_+ NO) oxidation conditions (McNeill et al., 2012) and the southeast has a mix of high-NO*_x_* and low-NO*_x_* (RO_2_+ HO_2_) conditions (Travis et al., 2016). In addition, abundant isoprene emissions can lead to substantial glyoxal concentrations. Modeling for the southeastern US indicates significant SOA can form from glyoxal (Marais et al., 2016; Pye et al., 2015; Knote et al., 2014; Li et al., 2016; Chan Miller et al., 2017). Implementation in models may require modifications to the gas-phase chemistry to specifically track glyoxal which may be lumped with other aldehydes (e.g., in CB05). Recent model studies do not find that a large SOA source from glyoxal is required to match observations, but more field measurements and laboratory studies, especially of the yield from isoprene oxidation and the aerosol uptake coefficient, are required to constrain the process.

#### 3.2.6 Cloud SOA

Results from SOAS and SEAC4RS indicate only a modest enhancement of OA due to cloud processing over the SE US, which was not statistically significant (Wagner et al., 2015). In addition, epoxide reactions in cloud droplets are predicted to lead to minor amounts of SOA due to the pH dependence of IEPOX hydrolysis (Fahey et al., 2017; McNeill, 2015).

#### 3.2.7 SOA from anthropogenic emissions

While the rural southeast is assumed to be dominated by SOA from biogenic precursors (which may be influenced by anthropogenic pollution) as a result of high modern carbon (Hidy et al., 2014), SOA from anthropogenic VOCs is known to play a role from fossil carbon measurements (~ 18 % at Centerville; Kim et al., 2015), but it is not directly apportioned otherwise. We note that since ~ 50 % of urban POA and 30 % of urban SOA is non-fossil (Zotter et al., 2014; Hayes et al., 2015); an urban fraction of ~ 28 % for the SOAS site is consistent with observations (Kim et al., 2015). This source is as large as most of the other individual sources discussed in this section and should not be neglected in modeling studies. A simple parameterization based on CO emissions (Hayes et al., 2015) may be adequate for incorporating this source in modeling studies and has shown good results for the southeastern US (Kim et al., 2015), but care should be taken to evaluate the CO emissions when using it.

#### 3.2.8 Surface network observations of organic aerosols

We list several caveats for the process of comparing model results to surface network observations. OC measurements from IMPROVE surface sites may be biased low in the summer due to evaporation of organic aerosols during the sample collection and handling (Kim et al., 2015). On the other hand, SEARCH measurements agree well with research community instruments in the Centerville site, such as AMS. Therefore the SEARCH data should be considered as the reference.

Decreases in mass concentrations of particulate sulfate and nitrate over the past decades are consistent with environmental policy targeting their gas-phase precursors, namely SO*_x_* and NO*_x_* emissions. Reductions in particulate organic carbon in the southeastern US over the past decade (Blanchard et al., 2016, 2013) are more difficult to reconcile because in the summertime it is predominantly modern and there is no control policy aimed at reducing biogenic VOCs. Decreased SO*_x_* (Kim et al., 2015; Xu et al., 2015b; Blanchard et al., 2013) and NO*_x_* emissions modulate the amount of organic aerosol formation through the gas-phase impacts described above and impacts on the absorbing medium amount (T. K. V. Nguyen et al., 2015; Attwood et al., 2014) and chemical composition.

In addition to sources and sinks of OA, attention should also be paid to the role of dry deposition of gases in determining mass loadings, as this process can have a large impact on model predictions and is very poorly constrained (Glasius and Goldstein, 2016; Knote et al., 2015).

#### 3.2.9 Climate-relevant properties

A motivating goal of the southeast studies was to examine PM mass measurements at the surface and satellite-measured AOD (aerosol optical depth) to facilitate improved prediction of the total aerosol loading. Aerosol mass aloft contributes to AOD (Wagner et al., 2015), and this complicates the relationship to surface concentrations. Relative humidity, vertical structure of the daytime PBL and aerosol liquid water (not measured by surface networks) influences remotely sensed AOD (Brock et al., 2016a, b; Kim et al., 2015; Nguyen et al., 2016). AOD is also complicated by aerosol composition. Attwood et al. (2014) finds that the steeper decrease in sulfate aerosol relative to organic from 2001 to 2013 has changed the hygroscopicity of SE US aerosol, leading to lower aerosol liquid water and thus lower optical extinction and AOD.

### 3.3 Model recommendations

Based upon the improved understanding outlined above, we make the following recommendations for the future modeling efforts:

1. There is high confidence that a pathway of SOA formation from isoprene epoxydiol (IEPOX) should be included in models. However, since many of the parameters needed to predict IEPOX-SOA are uncertain, further mechanistic studies are needed to address these uncertainties.
2. There is high confidence that models should include SOA formation from nitrate radical oxidation of monoterpenes (with or without explicit nitrate functionality). Sesquiterpenes and isoprene may also contribute SOA through nitrate radical oxidation, but the contribution is expected to be smaller.
3. More field measurements and laboratory studies, especially of the yield from isoprene oxidation and the aerosol uptake coefficient, are required to constrain the importance of glyoxal SOA.
4. There is high confidence that models should predict SOA from urban emissions with a parameterization that results in realistic concentrations. The non-fossil fraction of urban POA and SOA needs to be taken into account when interpreting modern carbon measurements.
5. Current SOA modeling efforts should be coupled with an up-to-date gas-phase chemistry to provide realistic concentrations for several important SOA precursors, including IEPOX, glyoxal, organic nitrates, etc.

### 3.4 Open questions

A number of open questions remain that would benefit from modeling studies:

1. What is the role of particle-phase organic nitrates in removing or recycling NO*_x_* from the system?
2. How much detail do models need to represent in terms of types of organic nitrate (ON)?
3. What are the formation mechanisms of highly oxygenated organics?
4. What anthropogenic sources of SOA are models missing?
5. What climate-relevant aerosol properties are needed in models? What are the controls over the presence and lifetime of condensed liquid water? What model and observational diagnostics serve as tests of our understanding?
6. What is the role of clouds in forming and processing organic aerosols?

## 4 Emissions

### 4.1 Background

Emission inventories are a critical input to atmospheric models, and reliable inventories are needed to design cost-effective strategies that control air pollution. For example, in the 1970s and 1980s, emission control strategies implemented under the Clean Air Act emphasized the control of anthropogenic VOC emissions over NO*_x_* (National Research Council, 2004). Despite large order-of-magnitude reductions in anthropogenic VOC emissions (Warneke et al., 2012), abatement of O_3_ was slow in many regions of the country. In the late 1980s, a large and underrepresented source of biogenic VOC emissions was identified (Trainer et al., 1987; Abelson, 1988; Chameides et al., 1988), putting into question the effectiveness of anthropogenic VOC emission control strategies to mitigate O_3_ nationally (Hagerman et al., 1997). Since the mid-1990s, large reductions in NO*_x_* emissions have resulted from (i) controls implemented at power plants (Frost et al., 2006), (ii) more durable three-way catalytic converters installed on gasoline vehicles (Bishop and Stedman, 2008) and (iii) more effective regulation of diesel NO*_x_* emissions from heavy-duty trucks (Yanowitz et al., 2000; McDonald et al., 2012). Emission reductions implemented on combustion sources have also been linked to decreases in organic aerosol concentrations observed in both California (McDonald et al., 2015) and the southeastern US (Blanchard et al., 2016). Though substantial progress has been made in improving scientific understanding of the major biogenic and anthropogenic sources of emissions contributing to air quality problems, some issues remain in current US inventories and are highlighted below.

The southeastern US is a region that has both large natural emissions and anthropogenic emissions. The accurate knowledge of biogenic emissions is key to understanding many of the processes that lead to ozone and aerosol formation. Previous studies suggest that MEGANv2.1 can estimate isoprene emissions that are twice as large compared with BEIS over the eastern US (Warneke et al., 2010; Carlton and Baker, 2011), but most global models using MEGANv2.1 do not show a significant bias of isoprene over the southeastern US (Mao et al., 2013; Millet et al., 2006). This is likely due to different land cover data being used in the regional and global applications of MEGAN. Validation of the various biogenic emission inventories was therefore one of the main science questions for the SAS studies.

The National Emissions Inventory developed by the US EPA is an inventory of air pollutants released every 3 years and commonly used in US-based air quality modeling studies. A recent modeling study reported that NO*_x_* emissions from mobile source emissions were overestimated by 51–70 % in the Baltimore–Washington, D.C., region (Anderson et al., 2014). Past studies have also found discrepancies in motor vehicle emission models used by the EPA to inform the NEI (Parrish, 2006; McDonald et al., 2012). Additionally, problems have been identified in estimates of NO*_x_*, VOC and methane emissions from US oil and gas development (Ahmadov et al., 2015; Pétron et al., 2014; Brandt et al., 2014). Some major oil and gas basins of note are located in the southeastern US, which were measured by aircraft during the SAS2013 studies. In contrast to mobile source and oil and gas emissions, power plant emissions of NO*_x_* and SO*_x_* are believed to be known with greater certainty since large stationary sources of emissions are continuously monitored. In addition to biogenic emission inventories, the data sets collected by the SAS2013 studies have provided an opportunity to assess the accuracy of anthropogenic emissions and their impacts on atmospheric chemistry.

The topic of model resolution, which involves the relationship between emissions and chemistry, is also key to interpreting model-observation comparisons. Regional-scale air quality models can be simulated at very high horizontal resolutions (e.g., 1 km and finer; Joe et al., 2014); however, typically they are run at coarser resolutions, such as at 12 km by 12 km (e.g., continental US; Gan et al., 2016) or 4 km by 4 km (e.g., urban scale; Kim et al., 2016b). The horizontal resolution of global chemistry models has significantly improved, with nesting being performed at horizontal resolutions as fine as 0.25° × 0.3125° (Travis et al., 2016). Coarse model resolutions can complicate evaluations with high spatial- and temporal-resolution measurements (e.g., from aircraft) of chemical constituents undergoing fast chemistry (e.g., isoprene, OH; Kaser et al., 2015). Sharp concentration gradients are observable from space for species with relatively short atmospheric lifetimes (e.g., nitrogen dioxide, formaldehyde and glyoxal) and potentially provide insights into the role of natural and anthropogenic emissions on air quality (Duncan et al., 2010; Russell et al., 2012; Lei et al., 2014). Lastly, some emission sources are described by large emission intensities (e.g., power plants and biomass burning), which result in elevated concentrations of emitted species downwind. A coarse model will artificially dilute these high emission fluxes (e.g., NO*_x_* and SO*_x_*) over a wider area, which could alter the chemical regime by which ozone (Ryerson et al., 1998, 2001) and secondary aerosols (Xu et al., 2015a) form.

### 4.2 Major relevant findings

#### 4.2.1 Biogenic emissions

Isoprene emissions measured by the NOAA P3, using the mixed boundary layer budget method, and NCAR/NSF C-130 and NASA DC-8 aircraft using direct eddy covariance flux measurements were within the wide range of observations reported by previous studies. The two methods of estimating isoprene emissions agreed within their uncertainties (Yu et al., 2017). Solar radiation and temperature measured by the aircraft along the flight tracks and available from regional model and assimilations (e.g., WRF, NLDAS-2) enabled estimation of emissions using models including BEIS3.12, BEIS3.13, MEGAN2.0, MEGAN2.1 with default land cover, MEGAN2.1 with revised land cover and MEGAN3. Isoprene emissions are highly sensitive to solar radiation and temperature, and biases in the values used to drive emission models can result in errors exceeding 40 %, complicating efforts to evaluate biogenic emission models. As has previously been noted in the southeastern US, MEGAN2.1 predicted isoprene emissions in the southeastern US were about twice as high as BEIS3.13. The measurements fall between the two models and are within the model and measurement uncertainties (Warneke et al., 2010). Isoprene mixing ratios were modeled with (a) WRF-Chem using BEIS and with (b) CAMx using MEGAN, and the results were consistent with the measurement–inventory comparison: WRF-Chem was biased low and CAMx biased high (Warneke et al., in preparation).

Land cover characteristics including leaf area index (LAI) and tree species composition data are also critical driving variables for BEIS and MEGAN isoprene and monoterpene emission estimates. Airborne flux measurements agreed well with MEGAN2.1 for landscapes dominated by southeastern oaks, which are high-isoprene-emitting tree species, but landscapes that had an overstory of non-emitters, with the high-isoprene emitters in the understory, showed emissions lower than expected by the model. The isoprene emission factor (EF) was linearly correlated with the high-isoprene-emitter plant species fraction in the land cover data set. This may indicate a need for models to include canopy vertical heterogeneity of the isoprene emitting fraction (Yu et al., 2017).

A simplification used in current biogenic emission models including BEIS3.13, BEIS3.6 and MEGAN2.1 is that all high-isoprene-emitting species are assigned the same isoprene emission factor. For example, all North American species of *Quercus* (oak), *Liquidambar* (sweetgum), *Nyssa* (tupelo), *Platanus* (sycamore), *Salix* (willow), *Robinia* (locust) and *Populus* (poplar and aspen) are assigned a single value based on the average of an extensive set of enclosure measurements conducted in North Carolina, California and Oregon in the 1990s (Geron et al., 2001). Earlier studies had reported isoprene emission factors for these tree species that ranged over more than an order of magnitude (Benjamin et al., 1996). Geron et al. (2001) showed that by following specific measurement protocols, including leaf cuvettes with environmental controls and ancillary physiological measurements such as photosynthesis, the variability dropped from over an order of magnitude to about a factor of 3. They concluded that this remaining variability was due at least as much to growth conditions as to species differences and so recommended that a single isoprene emission factor be used for all of these species. Recent aircraft flux measurements (Misztal et al., 2016; Yu et al., 2017) indicate that there is at least a factor of 2 difference in the isoprene emission factors of these species. This could be due to a genetic difference in emission capacity and/or differences in canopy structure. The aircraft measurements indicate that sweetgum and tupelo emission factors are similar to the value used in BESI3.13 and BEIS3.6, while the California oak emission factor is similar to that used in MEGAN2.1. The aircraft-based estimate of southeastern oak emission factors falls between the BEIS3.6 and MEGAN2.1 values. As a result, aircraft flux measurements in the southeastern US are higher than BEIS3.13 and BEIS3.6 and lower than MEGAN2.1. The MEGAN3 emission factor processor provides an approach for synthesizing available emission factor data and can be used to account for the emission rate variability observed by these aircraft flux studies (Guenther et al., 2018).

Modeling monoterpene emissions is even more challenging than isoprene emissions for reasons that include multiple emission processes (e.g., both light-dependent and light-independent emissions), stress-induced emission capability present in many plant species but not always expressed and the potential for enclosure measurements to dramatically overestimate emissions due to release of monoterpenes from damaged storage pools. The eddy covariance flux measurements on the NCAR/NSF C-130 are similar to the values estimated by MEGAN2.1 for needle leaf forests, considered to be high-emission regions, but are higher than the modeled monoterpene emissions from other landscapes (Yu et al., 2017). They conclude that unaccounted processes, such as floral and stress emissions, or sources such as non-tree vegetation may be responsible for the unexpectedly high monoterpene emissions observed by the aircraft.

During the experiment direct observations of fluxes for a variety of species from large aircraft were conducted, enabling a first direct estimate of fluxes over a regional domain (Wolfe et al., 2015; Yuan et al., 2015; Kaser et al., 2015). These data have the potential for enabling analyses of strengths and weaknesses of current emission and deposition schemes and their implementation within chemical transport models. Vertical flux profiles also contain information on the chemical production and loss rates, providing a new observational constraint on the processes controlling reactive gas budgets. An LES model was used to simulate isoprene, NO*_x_* and their variability in the boundary layer. The results showed good agreement between the measurements and the model. The atmospheric variability of isoprene, the altitude profile in the boundary layer of isoprene, and NO*_x_* mixing ratios and fluxes were well reproduced in the model, which was used to validate the eddy covariance and mixed boundary layer methods of estimating isoprene fluxes (Kim et al., 2016a; Wolfe et al., 2015).

#### 4.2.2 Anthropogenic emissions

Travis et al. (2016) utilizing the GEOS-Chem model report that NO*_x_* emissions are significantly overestimated by the NEI 2011 and suggest that mobile source and industrial emissions of NO*_x_* need to be lowered by 30–60 % to be consistent with aircraft measurements collected over the southeastern US during the SEAC4RS study. These results are consistent with modeling studies performed during the DISCOVER-AQ field campaign, which also found that the NEI 2011 overestimated NO*_x_* emissions (Anderson et al., 2014; Souri et al., 2016). However, a later study by Li et al. (2018) utilizing the AM3 model during the SENEX study suggests that overestimates in NEI 2011 NO*_x_* emissions may be smaller than reported in the Travis et al. study (~ 14 % vs. 30–60 %). McDonald et al. (2018) using WRF-Chem found mobile source emissions in the NEI 2011 to be overestimated by ~ 50 % and a factor of 2.2 for NO*_x_* and CO, respectively, when evaluated with SENEX aircraft measurements. Due to rapidly declining trends in vehicle emissions (McDonald et al., 2013, 2012), some of the emissions overestimate was attributed to utilizing a 2011 inventory in 2013 model simulations. However, roadside measurements of vehicular exhaust also suggest systematic overestimates in emission factors used by the EPA’s vehicle emissions model (MOVES), likely contributing to the consistent reporting to date of overestimated mobile source NO*_x_* emissions (Anderson et al., 2014; Souri et al., 2016; Travis et al., 2016). When NO*_x_* emissions were reduced from mobile sources by this amount, model predictions of O_3_ over the southeastern US were improved both for mean concentrations and O_3_ extreme days (McDonald et al., 2018), consistent with modeling by Li et al. (2018) demonstrating the sensitivity of O_3_ to NO*_x_* emissions in the southeastern US over the 2004–2013 timespan.

Along with other aircraft field campaigns and tall tower measurements in the Upper Midwest, data from the SENEX study was used to assess anthropogenic emissions of VOCs in the NEI and a global inventory (RETRO). L. Hu et al. (2015) found that RETRO consistently overestimates US emissions of C6–C8 aromatic compounds by factors of 2–4.5; the NEI 2008 overestimates toluene by a factor of 3 but is consistent with top-down emission estimates for benzene and C8 aromatics. The study also suggests that East Asian emissions are an increasingly important source of benzene concentrations over the US, highlighting the importance of long-range transport on US air quality as domestic sources of emissions decline (Warneke et al., 2012).

Two studies have quantified top-down emissions of oil and gas operations, derived from aircraft measurements for VOCs and methane from SENEX P-3 data (Peischl et al., 2015; Yuan et al., 2015). The oil and gas regions measured during SENEX account for half of the US shale gas production, and loss rates of methane to the atmosphere relative to production were typically lower than prior assessments (Peischl et al., 2015). Yuan et al. (2015) explored the utility of eddy-covariance flux measurements on SENEX and NO-MADSS aircraft campaigns and showed that methane emissions were disproportionately from a subset of higher emitting oil and gas facilities. Strong correlations were also found between methane and benzene, indicating that VOCs are also emitted in oil and gas extraction. High wintertime O_3_ has been found in the Uintah Basin, UT (Ahmadov et al., 2015; Edwards et al., 2014), though it is unclear at this time how significant oil and gas emissions of VOCs could be in an isoprene-rich source region on tropospheric O_3_ formation. Future atmospheric modeling efforts of oil and gas emissions are needed.

During the SENEX and SEAC4RS studies, research aircraft measured agricultural fires over the southeast. Liu et al. (2016) reported emission factors of trace gases, which were consistent with prior literature. In general, the authors found emissions of SO_2_, NO*_x_* and CO from agricultural fires to be small relative to mobile sources (< 10 %). However, within fire plumes, rapid O_3_ formation was observed, indicating potential air quality impacts on downwind communities. To represent the impact of biomass burning, air quality models need improved treatments of initial VOC and NO*_x_* emissions and near-source chemistry. Sub-grid parameterizations, based on detailed models like the Aerosol Simulation Program (ASP; Alvarado and Prinn, 2009) and which incorporate gas-phase chemistry, inorganic and organic aerosol thermodynamics, and evolution of aerosol size distribution and optical properties, could improve coarse model representations of chemistry near biomass burning plumes. Zarzana et al. (2017) investigated enhancements of glyoxal and methylglyoxal relative to CO from agricultural fires and report that global models may overestimate biomass burning emissions of glyoxal by a factor of 4. This highlights large uncertainties and variability in fire emissions and a need for additional observational constraints on inventories and models.

### 4.3 Model recommendations and future work

1. In the southeastern US, isoprene emissions are so large that they influence most atmospheric chemistry processes. Users of model simulations using the different isoprene inventories have to be aware of the differences. For example, OH and isoprene concentrations are anti-correlated (Kim et al., 2015) and model simulations using BEIS will potentially have higher OH than simulations using MEGAN and chemistry will proceed at different rates. In addition, modeled products from isoprene oxidation in the gas and particle phase will be different. Isoprene-derived SOA or secondary CO in the southeastern US can vary by a factor of 2 between the two inventories.
2. For future work, BEIS3.6 is now available and needs to be evaluated using the methods described here.
3. The MEGAN3 emission factor processor can be used to synthesize the available emission factor estimates from SAS and other studies. A beta version of the MEGAN3 emission factor processor and MEGAN3 model processes is available and should be evaluated.
4. A revised NO*_x_* emissions inventory is needed to improve air quality models for O_3_, especially in the southeastern US where O_3_ is sensitive to changes in NO*_x_* emissions. Anthropogenic emissions of NO*_x_* in the NEI 2011 may be overestimated by 14–60 % in the southeastern US during the SAS2013 study time period (Travis et al., 2016; Li et al., 2018).

## 5 Chemistry–climate interactions

### 5.1 Background

Interactions between atmospheric chemistry and climate over the southeastern United States are not well quantified. The dense vegetation and warm temperatures over the southeast result in large emissions of isoprene and other biogenic species. These emissions, together with anthropogenic emissions, lead to annual mean aerosol optical depths of nearly 0.2, with a peak in summer (Goldstein et al., 2009). The climate impacts of US aerosol trends in the southeast due to changing anthropogenic emissions are under debate (e.g., Leibensperger et al., 2012a, b; Yu et al., 2014). Climate change can, in turn, influence surface air quality, but even the sign of the effect is unknown in the southeast (Weaver et al., 2009). Part of this uncertainty has to do with complexities in the mechanism of isoprene oxidation, the details of which are still emerging from laboratory experiments and field campaigns (Liao et al., 2015; Fisher et al., 2016; Marais et al., 2016). In addition, the influence of day-to-day weather on surface ozone and particulate matter (PM_2.5_) has not been fully quantified, and climate models simulate different regional climate responses. Resolving these uncertainties is important, as climate change in the coming decades may impose a “climate penalty” on surface air quality in the southeast and elsewhere (Fiore et al., 2015).

### 5.2 Key science issues and recent advances

We describe recent advances in four areas related to chemistry–climate interactions in the southeast.

#### 5.2.1 Seasonality and trends in aerosol loading in the southeast

Using satellite data, Goldstein et al. (2009) diagnosed summertime enhancements in AOD of 0.18 over the southeast, relative to winter, and hypothesized that secondary organic aerosol from biogenic emissions accounts for this enhancement. Goldstein et al. (2009) further estimated a regional surface cooling of −0.4 W m^−2^ in response to annual mean AOD over the southeast. These findings seemed at first at odds with surface PM_2.5_ measurements, which reveal little seasonal enhancement in summer. Using SEAC4RS measurements and GEOS-Chem, Kim et al. (2015) determined that the relatively flat seasonality in surface PM_2.5_ can be traced to the deeper boundary layer in summer, which dilutes surface concentrations.

In response to emission controls, aerosol loading over the southeast has declined in recent decades. For example, wet deposition fluxes of sulfate decreased by as much as ~ 50 % from the 1980s to 2010 (Leibensperger et al., 2012a). Over the 2003–2013 time period, surface concentrations of sulfate PM_2.5_ declined by 60 %. Organic aerosol (OA) also declined by 60 % even though most OA appears to be biogenic and there is no indication of a decrease in anthropogenic sources (Kim et al., 2015). Model results suggest that the observed decline in OA may be tied to the decrease in sulfate, since OA formation from biogenic isoprene depends on aerosol water content and acidity (Marais et al., 2016, 2017). Consistent with these surface trends, 550 nm AOD at AERONET (Aerosol Robotic Network) sites across the southeast has also decreased, with trends of −4.1 % a^−1^ from 2001 to 2013 (Attwood et al., 2014). Xing et al. (2015a) reported a roughly −4 % decrease in remotely sensed AOD across the eastern United States, as measured by the Moderate Resolution Imaging and Spectroradiometer (MODIS) on board Terra and Aqua. These large declines could potentially have had a substantial impact on regional climate, both through aerosol–radiation interactions and aerosol–cloud interactions.

#### 5.2.2 Contribution of aerosol trends to the US “warming hole”

**Figure 3:**
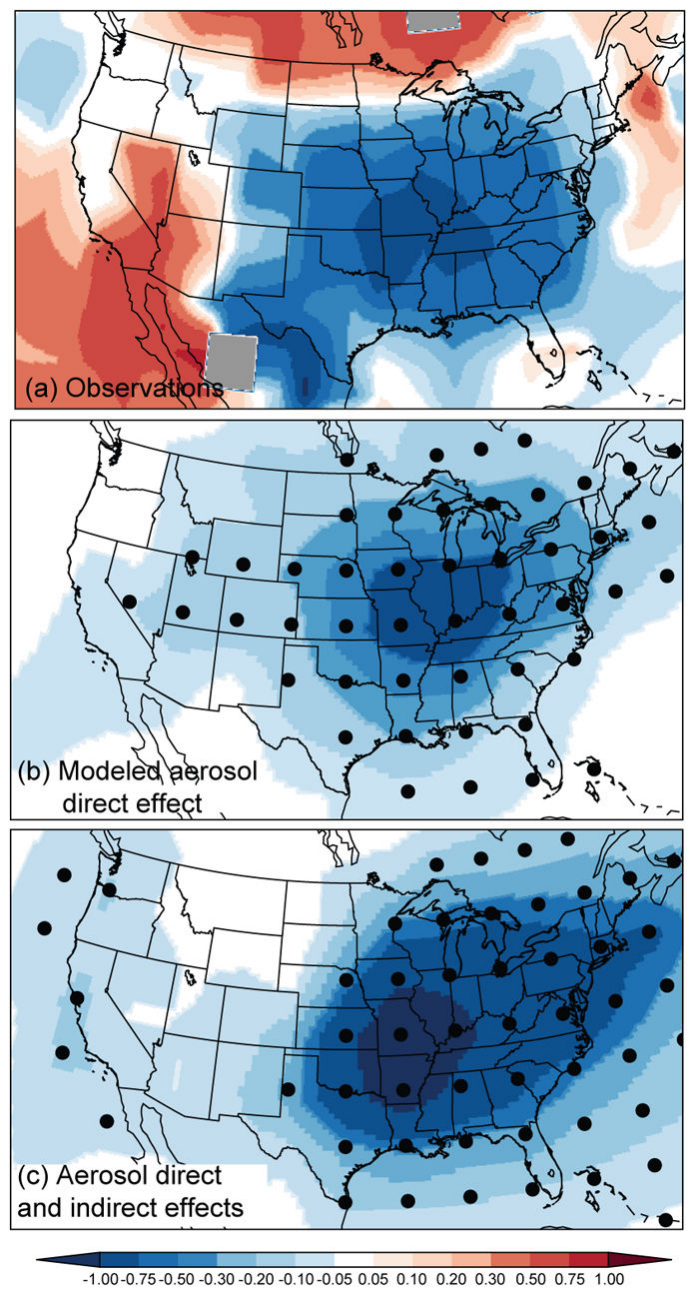
Observed difference in surface air temperature between 1930 and 1990 **(a)** and modeled effect of US anthropogenic aerosol sources on surface air temperatures for the 1970–1990 period when US aerosol loading was at its peak (**b** and **c**; Leibensperger et al., 2012a). Observations are from the NASA GISS Surface Temperature Analysis (GISTEMP; http://data.giss.nasa.gov/gistemp/). Model values represent the mean difference between 5-member ensemble GCM simulations including vs. excluding US anthropogenic aerosol sources and considering the aerosol direct only **(b)** and the sum of direct and indirect effects **(c)**. In **(b)** and **(c)**, dots indicate differences significant at the 95th percentile.

Even as global mean temperatures rose over the 20th century in response to increasing greenhouse gases, significant cooling occurred over the central and southeastern United States. This cooling, referred to as the US warming hole (Pan et al., 2004), has been quantified in several ways. For example, Fig. 3 shows that annual mean temperatures across the southeast decreased by ~ 1 °C during the 1930–1990 time-frame (Capparelli et al., 2013). A different temperature metric, the 20-year annual return value for the hot tail of daily maximum temperatures, decreased by 2° from 1950 to 2007 (Grotjahn et al., 2016). Over a similar time frame, Portmann et al. (2009) diagnosed declines in maximum daily temperatures in the southeast of 2–4° per decade, with peak declines in May–June, and linked these temperature trends with regions of high climatological precipitation. Since the early 2000s, the cooling trend has appeared to reverse (Meehl et al., 2015).

The causes of the US warming hole are not clear. Most freely running climate models participating in the Coupled Model Intercomparison Project (CMIP5) cannot capture the observed 20th century temperature trends over the southeast (Knutson et al., 2013; Kumar et al., 2013; Sheffield et al., 2013); this failure likely arises from either model deficiency or natural variability not included in the simulations. Indeed, several studies have argued that naturally occurring oscillations in sea surface temperatures (SSTs) influenced the large-scale cooling in the southeast (Robinson et al., 2002; Kunkel et al., 2006; Meehl et al., 2012; Weaver, 2013; Mascioli et al., 2017). Kumar et al. (2013), for example, linked the June–July–August indices of the Atlantic Multidecadal Oscillation (AMO) to annual mean temperatures across the eastern US for the 1901–2004 period. Mauget and Cordero (2014), however, pointed out inconsistencies in these two time series, with the AMO index sometimes lagging temperature changes. A recent study has argued that the transition of the Interdecadal Pacific Oscillation (IPO) phase from positive to negative in the late 1990s may have triggered a reversal of the warming hole trend (Meehl et al., 2015).

The cool period in the southeast coincided with heavy aerosol loading over the region, and several studies have suggested that trends in aerosol forcing may have also played a role in driving the US warming hole. For example, Leibensperger et al. (2012a, b) found that the regional radiative forcing from anthropogenic aerosols led to a strong regional climate response, cooling the central and eastern US by 0.5–1.0° from 1970 to 1990 (Fig. 3), with the strongest effects on maximum daytime temperatures in summer and autumn. In that study, the spatial mismatch between maximum aerosol loading and maximum cooling could be partly explained by aerosol outflow cooling the North Atlantic, which strengthened the Bermuda High and increased the flow of moist air into the south-central United States. Another model study diagnosed positive feedbacks between aerosol loading, soil moisture and low cloud cover that may amplify the local response to aerosol trends (Mickley et al., 2012). The strength of such positive feedbacks may vary regionally, yielding different sensitivities in surface temperature to aerosol forcing.

The cool period in the southeast coincided with heavy aerosol loading over the region, and several studies have suggested that trends in aerosol forcing may have also played a role in driving the US warming hole. For example, Leibensperger et al. (2012a, b) found that the regional radiative forcing from anthropogenic aerosols led to a strong regional climate response, cooling the central and eastern US by 0.5–1.0° from 1970 to 1990 (Fig. 3), with the strongest effects on maximum daytime temperatures in summer and autumn. In that study, the spatial mismatch between maximum aerosol loading and maximum cooling could be partly explained by aerosol outflow cooling the North Atlantic, which strengthened the Bermuda High and increased the flow of moist air into the south-central United States. Another model study diagnosed positive feedbacks between aerosol loading, soil moisture and low cloud cover that may amplify the local response to aerosol trends in the eastern US, including the southeast (Mickley et al., 2012). The strength of such positive feedbacks may vary regionally, yielding different sensitivities in surface temperature to aerosol forcing. More recent modeling studies, however, have generated conflicting results regarding the role of aerosols in driving the warming hole. For example, the model study of Mascioli et al. (2016) reported little sensitivity in southeast surface temperatures to external forcings such as anthropogenic aerosols or even greenhouse gases. In contrast, Banerjee et al. (2017) found that as much of 50 % of the observed 1950–1975 summertime cooling trend in the southeast could be explained by increasing aerosols. Examining multi-model output, Mascioli et al. (2017) concluded that aerosols accounted for just 17 % of this cooling trend in summer. These contrasting model results point to the challenges in modeling climate feedbacks, such as those involving cloud cover or soil moisture.

These early model studies have been accompanied by more observationally based efforts to link trends in surface temperature to aerosol loading. A key first step is to determine whether changes in surface solar radiation are related to changes in aerosol loading. Measurements from the Surface Radiation network (SURFRAD) reveal increases of +0.4 Wm^−2^ a^−1^ in total surface solar radiation across the east during 1995–2010 (Gan et al., 2014). An attempt to reproduce the trend in total surface radiation with a regional chemistry–climate model found a reasonable match with observations over the east when aerosol–radiation interactions were included (Xing et al., 2015a). Most of the observed increase in surface solar radiation, however, appears due to increasing diffuse radiation, at odds with the decline in AOD, which should instead increase direct radiation (Gan et al., 2015, 2014). Using satellite data and assimilated meteorology, Yu et al. (2014) showed that trends in spatially averaged AOD and cloud optical depth declined over the 2000–2011 time period over the eastern US, while daily maximum temperatures and shortwave cloud forcing increased. These opposing trends suggest that aerosol–cloud interactions may have influenced the observed ~ 1° warming trend in the southeast over this 10-year time period, with the decline in anthropogenic aerosols driving a decrease in cloud cover and a rise in surface temperatures. Yu et al. (2014) confirmed this hypothesis using a chemistry–climate model. In contrast, the observational study of Tosca et al. (2017), which also relied on satellite AOD, pointed to aerosol–radiation interactions as the driver of surface temperature trends in the southeast. Analysis of ground-based observations in Mississippi, however, found little covariability between AOD and clear-sky solar radiation at the surface, casting doubt on the importance of aerosol–radiation interactions in driving the observed cooling in this region (Cusworth et al., 2017).

Continued improvements of PM_2.5_ air quality in the southeast may further influence regional climate. Y. Lee et al. (2016) projected a warming of about +0.5 Wm^−2^ over the eastern US, including the southeast, over the 2000–2030 timeframe due to anticipated improvements in air quality and the associated reduction in AOD. Xing et al. (2015b) have pointed out that an overlooked beneficial effect of aerosol reduction is increased ventilation of surface air, a positive feedback that leads to further decline in surface PM_2.5_ concentrations. The feedback arises from changes in the temperature profile, with warmer temperatures at the surface and cooler temperatures aloft, which together enhance atmospheric instability and ventilation as aerosol-induced cooling is reduced. The feedback may lead to unexpected health benefits of clearing PM_2.5_ pollution (Xing et al., 2016).

#### 5.2.3 Influence of meteorology on surface air quality in the southeast

Pollution episodes in the southeastern United States are correlated with high temperatures, low wind speeds, clear skies and stagnant weather (Camalier et al., 2007; Jacob and Winner, 2009). The spatial extent of the Bermuda High also plays a role in modulating air quality in the southeast (Zhu and Liang, 2013).

Fu et al. (2015) used models and observations to examine the sensitivity of August surface ozone in the southeast to temperature variability during 1988–2011. This study finds that warmer temperatures enhance ozone by increasing biogenic emissions and accelerating photochemical reaction rates. However, variability in ozone advection into the region may also explain much of the variability of surface ozone, with possibly increased advection occurring during the positive phase of the Atlantic Multidecadal Oscillation. Applying empirical orthogonal functions (EOF) analysis to observed ozone, Shen et al. (2015) determined that the sensitivity of surface ozone in the southeast can be quantified by the behavior of the west edge of the Bermuda High. Specifically, for those summers when the average position of the west edge is located west of ~ 85.4° W, a westward shift in the Bermuda High west edge increases ozone in the southeast by 1 ppbv deg^−1^ in longitude. For all summers, a northward shift in the Bermuda High west edge increases ozone over the entire eastern United States by 1–2 ppbv deg^−1^ in latitude.

The influence of meteorology on PM_2.5_ in the southeast is not well quantified. Tai et al. (2010) found that observed sulfate and OC concentrations increase with increasing temperature across the region due to faster oxidation rates and the association of warm temperatures with stagnation and biogenic and fire emissions. Nitrate PM_2.5_, however, becomes more volatile at higher temperatures and decreases with temperature. Using local meteorology, however, Tai et al. (2010) could explain only about 20–30 % of PM_2.5_ daily variability in the southeast. Both Thishan Dharshana et al. (2010) and Tai et al. (2012b) diagnosed a relatively weak effect of synoptic-scale weather systems on PM_2.5_ air quality in the southeast, especially in the deep south. Shen et al. (2017), however, extended the statistical studies of Tai et al. (2012a, b) by taking into account not just the local influences of meteorology on PM_2.5_ air quality but also the relationships between local PM_2.5_ and meteorological variables in the surrounding region. These authors developed a statistical model that explains 30–50 % of PM_2.5_ monthly variability in the southeast. Shen et al. (2017) further reported that many atmospheric chemistry models may underestimate or even fail to capture the strongly positive sensitivity of monthly mean PM_2.5_ to surface temperature in the eastern United States, including the southeast, in summer. In GEOS-Chem, this underestimate can be traced to the overly strong tendency of modeled low cloud cover to decrease as temperatures rise (Shen et al., 2017).

#### 5.2.4 Effects of future climate change on southeast air quality

Emissions of US pollution precursors are expected to decline in coming decades (Lamarque et al., 2013; Fiore et al., 2015), which may offset any potential climate penalty. Background ozone, however, may increase due to increasing methane (West et al., 2012). A major challenge in quantifying the future trends in surface air quality is our lack of knowledge in temperature-dependent isoprene emissions and photochemistry (Achakulwisut et al., 2015).

Using a regional chemistry–climate model, Gonzalez-Abraham et al. (2015) found that daily maximum 8 h average (MDA8) ozone concentrations in the southeast would likely increase by 3–6 ppbv by the 2050s due solely to climate change and land use change. Changes in anthropogenic emissions of ozone precursors such as methane could further enhance MDA8 ozone in the southeast by 1–2 ppbv. Rieder et al. (2015), however, determined that large areas of the southeast would experience little change in surface ozone by the 2050s, but that study neglected the influence of warming temperatures on biogenic emissions. Shen et al. (2016) developed a statistical model using extreme value theory to estimate the 2000–2050 changes in ozone episodes across the United States. Assuming constant anthropogenic emissions at the present level, they found an average annual increase in ozone episodes of 2.3 days (> 75 ppbv) across the United States by the 2050s, but relatively little change in the southeast. In fact, a key result of this work is the relative insensitivity of ozone episodes to temperature in the southeast. However, Zhang and Wang (2016) have suggested that warmer and drier conditions in the southeast future atmosphere could extend the ozone season, leading to ozone episodes in October.

Model studies differ on the effects of future climate change on PM_2.5_ in the southeast. Tai et al. (2012a, b) analyzed trends in meteorological modes from an ensemble of climate models and found only modest changes in annual mean PM_2.5_ (±0.4 μg m^−3^) by the 2050s in the southeast, relative to the present-day. Using a single chemistry–climate model, Day and Pandis (2015) calculated significant increases of ~3.6 μg m^−3^ in July mean PM_2.5_ along the Gulf coast by the 2050s and attributed these increases to a combination of decreased rain-out, reduced ventilation and increased biogenic emissions. Building on the statistical model of Tai et al. (2012a,b), Shen et al. (2017) found that PM_2.5_ concentrations in the southeast could increase by 0.5–1.0 μg m^−3^ by 2050 on an annual basis and as much as 2.0–3.0 μg m^−3^ in summer, assuming anthropogenic emissions remained at present-day levels. These authors found that the driver for these increases was rising surface temperature, which influences both biogenic emissions and the rate of sulfate production.

### 5.3 Open questions

Unresolved issues in chemistry–climate interactions in the southeast include the following:

1. What is the impact of aerosols on the regional climate of the southeast? What role do feedbacks play, including feedbacks involving cloud cover, soil moisture and boundary layer height? Did land use changes play a role in the southeast warming hole? How will changing aerosol composition affect regional climate? Can we reconcile observed trends in insolation and aerosols? Can we use observed weekly cycles in temperature or precipitation to probe possible aerosol effects on regional climate (Forster and Solomon, 2003; Bell et al., 2008; Bäumer et al., 2008; Daniel et al., 2012)?
2. What caused the US warming hole? Is the observed cooling over the southeast partly due to natural variability of North Atlantic SSTs? Do aerosol changes induce changes in the North Atlantic SSTs that feed back on the southeastern US? Has the warming hole ended and made the central and southeastern United States more vulnerable to high temperatures and drought?
3. What limits model skill in simulating the variability of surface pollution in the southeast? Can we capture the observed effects of the Bermuda High or the AMO on surface air quality?
4. How will air quality in the southeast change in the future? Do current model weaknesses in simulating present-day ozone and PM_2.5_ daily or seasonal variability limit our confidence in future projections?

### 5.4 Model recommendations

We recommend the following approaches for studies involving chemistry–climate interactions in the southeastern US.

1. Take advantage of findings from the 2013 measurement campaigns.
  For aerosol, such findings include information on composition, hygroscopicity, lifetime, aerosol–cloud interactions, optical properties and the mechanism of SOA formation. Modelers should also take advantage of new information on isoprene emission flux and oxidation mechanisms.
2. Link 2013 results with findings from previous measurement campaigns and with long-term in situ and satellite data.
3. Work to apply best practices, including standard statistical tests, to chemistry–climate studies.
  Modelers need to consider the statistical significance of observed trends and perform ensemble simulations for robust statistics. The auto-correlation of the variables under investigation should be examined. Comparison of observed trends with samples of internal climate variability from model control runs, as in (Knutson et al., 2013), may be a useful approach, and modelers should acknowledge that observations may represent an outlier of unforced variability.
4. Benchmark chemistry–climate models in a way that is useful for chemistry–climate studies.
  For the southeast, modelers should consider testing the following model properties:
  - Sensitivity of surface air quality to synoptic weather systems, including the westward extent of the Bermuda High and cold front frequency.
  - Sensitivity of surface air quality to local meteorological variables and isoprene emissions on a range of temporal scales.
  - Sensitivity of soil moisture and cloud cover to changing meteorology and the consequences for regional climate and air quality.

## 6 Summary

The primary purpose of this work is to improve model representation of fundamental processes over the southeastern US. We summarize the modeling recommendations as follows.

### Gas-phase chemistry

(1) Up-to-date “standard” chemical mechanisms represent OH chemistry well over the observed range of NO*_x_* concentrations. Detailed mechanisms based on recent laboratory chamber studies (mostly at Caltech) and theoretical studies (Leuven) for isoprene chemistry result in predicted OH that is in reasonable agreement with observations. Condensed mechanisms that approximate these details are expected to do the same. (2) Given the large emissions and high chemical reactivity of isoprene, its chemistry should be treated fairly explicitly, including more detail than for most other hydrocarbons. (3) NO_3_ chemistry contributes significantly to both VOC oxidation and aerosol production. (4) The regions of peak NO*_x_* and BVOC emissions are not collocated. As a result, the model resolution can impact the predictions.

### Organic aerosol

(1) There is high confidence that a pathway of SOA formation from isoprene epoxydiol (IEPOX) should be included in models. However, since many of the parameters needed to predict IEPOX-SOA are uncertain, further mechanistic studies are needed to address these uncertainties. (2) There is high confidence that models should include SOA formation from nitrate radical oxidation of monoterpenes (with or without explicit nitrate functionality). Sesquiterpenes and isoprene may also contribute SOA through nitrate radical oxidation, but the contribution is expected to be smaller. (3) More field measurements and laboratory studies, especially of the yield from isoprene oxidation and the aerosol uptake coefficient, are required to constrain the importance of glyoxal SOA. (4) There is high confidence that models should include SOA from urban emissions with a parameterization that results in realistic concentrations.

### Natural and anthropogenic emissions

(1) Biogenic emissions from BEIS are generally lower, and those from MEGAN generally higher, than from measurements for all campaigns. (2) Observations confirm a rapid decrease in ozone precursor emissions over past few decades. Thus, use of the correct scaling of anthropogenic emissions for a particular year is important for accurate simulations. (3) National Emissions Inventory 2011 likely overestimates NO*_x_* emissions in the study area from mobile sources that use fuel-based estimates.

### Climate and chemistry interactions

(1) Annual mean temperatures during the 1930–1990 timeframe decreased by ~ 1 ° C over the central and southeastern United States. Several studies have argued that patterns of sea surface temperatures in the North Atlantic may have caused this large-scale cooling. Trends in aerosol forcing may have also played a role. (2) Pollution episodes in the southeastern United States are correlated with high temperatures, low wind speeds, clear skies and stagnant weather. Surface air quality over the southeastern US may be to some extent modulated by large-scale circulations, such the Bermuda High or Atlantic Multi-decadal Oscillation.

## Figures and Tables

**Table 1:** A subset of model evaluations for SAS observations (till 2017).

Model name	Model type	References	Targeted species	Major findings
F0AM	0-D	Feiner et al. (2016)	OH, HO_2_, OH reactivity	Measured and modeled OH agree well.
Box model	0-D	B. H. Lee et al. (2016)	Speciated organic nitrates	Particle-phase organic nitrates are an important component in organic aerosols but could have a short particle-phase lifetime.
F0AM	0-D	Wolfe et al. (2016)	HCHO	Current models accurately represent early-generation HCHO production from isoprene but under-predict a persistent background HCHO source.
F0AM	0-D	Kaiser et al. (2016)	OH reactivity	Missing OH reactivity is small.
F0AM	0-D	Marvin et al. (2017)	HCHO	Model HCHO–isoprene relationships are mechanism dependent. Condensed mechanisms (esp. CB6r2) can perform as well as explicit ones with some modifications.
ISORROPIA	0-D	Weber et al. (2016); Guo et al. (2015)	Aerosol acidity	Submicron aerosols are highly acidic in the southeastern US.
MXLCH	1-D	Su et al. (2016)	Isoprene, HCHO, MVK, MACR, organic nitrates, OH, HO_2_	Diurnal evolution of O_3_ is dominated by entrainment. Diurnal evolution of isoprene oxidation products are sensitive to the NO : HO_2_ ratio.
GEOS-Chem	3-D	Fisher et al. (2016)	Organic nitrates	Updated isoprene chemistry, new monoterpene chemistry and particle uptake of RONO_2_. RONO_2_ production accounts for 20 % of the net regional NO*_x_* sink in the southeast in summer.
GEOS-Chem	3-D	Travis et al. (2016)	NO*_x_*, ozone	NEI NO*_x_* emissions from mobile and industrial sources reduced by 30–60 %. The model is still biased high by 6– 14 ppb relative to observed surface ozone.
GEOS-Chem	3-D	Zhu et al. (2016)	HCHO	GEOS-Chem used as a common intercomparison platform among HCHO aircraft observations and satellite data sets of column HCHO. The model shows no bias against aircraft observations.
GEOS-Chem	3-D	Kim et al. (2015)	Organic and inorganic aerosols	GEOS-Chem used as a common platform to interpret observations of different aerosol variables across the southeast. Surface PM_2.5_ shows far less summer-to-winter decrease than AOD.
GEOS-Chem	3-D	Chan Miller et al. (2017)	Glyoxal, HCHO	New chemical mechanism for glyoxal formation from isoprene. Observed glyoxal and HCHO over the southeast are tightly correlated and provide redundant proxies of isoprene emissions.
GEOS-Chem	3-D	Marais et al. (2016)	IEPOX, organic aerosols	New aqueous-phase mechanism for isoprene SOA formation. Reducing SO_2_ emissions in the model decreases both sulfate and SOA by similar magnitudes.
GEOS-Chem	3-D	Silvern et al. (2017)	Aerosol acidity	Sulfate aerosols may be coated by organic material, preventing NH_3_ uptake.
GFDL AM3	3-D	Li et al. (2016)	Glyoxal, HCHO	Gas-phase production of glyoxal from isoprene oxidation represents a large uncertainty in quantifying its contribution to SOA.
GFDL AM3	3-D	Li et al. (2018)	Organic nitrates, ozone	Reactive oxidized nitrogen species, including NO*_x_*, PAN and HNO_3_, decline proportionally with decreasing NO*_x_* emissions in the southeastern US.
CMAQ	3-D	Pye et al. (2015)	Terpene nitrates	Monoterpene + NO_3_ reactions responsible for significant NO*_x_* -dependent SOA. Magnitude of SOA dependent on assumptions regarding hydrolysis.
Box model with CMAQ/simple-GAMMA algorithms	0-D	Budisulistiorini et al. (2017); Budisulistiorini et al. (2015)	IEPOX, SOA	Sulfate, through its influence on particle size (volume) and rate of particle-phase reaction (acidity), controls IEPOX uptake at Look Rock (LRK).
CMAQ	3-D	Pye et al. (2017)	Aerosol liquid water, water soluble organic carbon (WSOC)	Aerosol water requires accurate organic aerosol predictions as models considering only water associated with inorganic ions will underestimate aerosol water. Gas-phase WSOC, including IEPOX + glyoxal + methylglyoxal, is abundant in models.
CMAQ	3-D	Fahey et al. (2017)	Cloud-mediated organic aerosol	Cloud-processing of IEPOX increased cloud-mediated SOA by a modest amount (11 to 18 % at the surface in the eastern US)
CMAQ	3-D	Murphy et al. (2017)	Organic aerosol from combustions sources	At the Centerville (CTR) site, organic aerosol predictions are not very sensitive to assumptions (volatility, oxidation) for combustion-derived organic aerosol.
CMAQ	3-D	Baker and Woody (2017)	Ozone, PM2.5	Single-source impacts of a coal fired power plant, including the contribution to secondary pollutants, can be estimated from a 3-D CTM.
AIOMFAC, CMAQ	0-D/3-D	Pye et al. (2018)	Inorganic aerosol, semivolatile species	Thermodynamic models are consistent with SEARCH and MARGA measured ammonium sulfate at CTR. Organic– inorganic interactions can cause small decreases in acidity and increased partitioning to the particle for organic species with O : C > 0.6.
WRF-Chem	3-D	McDonald et al. (2018)	NO*_x_*, CO, ozone	Mobile source NO*_x_* and CO emissions overestimated by 50 % and factor of 2.2, respectively. Model surface O_3_ improves with reduced mobile source NO*_x_* emissions.
NCAR LES	3-D	Kim et al. (2016a)	Isoprene, OH	Turbulence impacts isoprene-OH reactivity, and effect depends on NO*_x_* abundance.

## Appendix A: Glossary of acronyms

AIOMFAC: Aerosol Inorganic–Organic Mixtures Functional groups Activity Coefficients model
AM3: the atmospheric component of the GFDL coupled climate model CM3
AMS: aerosol mass spectrometer
AMO: Atlantic Multidecadal Oscillation
AOD: aerosol optical depth
BBOA: biomass burning OA
BEIS: Biogenic Emission Inventory System
BVOCs: biogenic volatile organic compounds
CAMx: Comprehensive Air Quality Model with Extensions
CMAQ: Community Multiscale Air Quality Model
EF: emission factor
F0AM: Framework for 0-D Atmospheric Modeling
GFDL: Geophysical Fluid Dynamics Laboratory
HOA: hydrocarbon-like OA
IEPOX: isoprene epoxydiol
IMPROVE: Interagency Monitoring of Protected Visual Environments visibility monitoring network
LAI: leaf area index
LES: Large-eddy simulation
LO-OOA: less-oxidized oxygenated OA
MACR: methacrolein
MARGA: Monitor for Aerosols and Gases in Air
MEGAN: Model of Emissions of Gases and Aerosols from Nature
MO-OOA: more-oxidized oxygenated OA
MVK: methyl vinyl ketone
MXLCH: mixed-layer chemistry model
NEI: National Emissions Inventory
NOAA: National Oceanic and Atmospheric Administration
NOMADSS: Nitrogen, Oxidants, Mercury and Aerosol Distributions, Sources and Sinks aircraft campaign, which took place during June–July 2013 with the NSF/NCAR C-130 aircraft
OA: organic aerosol
OC: organic carbon
OM: organic matter
PAN: peroxyacetyl nitrate
PMF: positive matrix factorization
POA: primary organic aerosol
SAS: Southeast Atmosphere Studies
SEAC4RS: Studies of Emissions, Atmospheric Composition, Clouds and Climate Coupling by Regional Surveys aircraft campaign, which took place during August–September 2013 with NASA DC-8 and ER-2 aircraft
SEARCH: Southeastern Aerosol Research and Characterization Network
SENEX: Southeast Nexus of air quality and climate campaign
S / IVOCs: semivolatile / intermediate volatility organic compounds
SOA: secondary organic aerosols
SOAS: the Southern Oxidant and Aerosol Study ground-based campaign, which took place during June–July 2013 near Brent, Alabama
SURFRAD: Surface Radiation Budget Network
VBS: volatility basis set
WRF-Chem: Weather Research and Forecasting with Chemistry model
